# A New Hypothesis of the Cause of Diabetes Mellitus

**Published:** 1820-02

**Authors:** 


FOR THE LONDON MEDICAL AND PHYSICAL JOURNAL.

### *A new Hypothesis of the Cause of Diabetes Mellitus.* By Professor HUFELAND.

DIABETES Mellitus appears to be progressively increasing in frequency. We have, within a few years, had occasion to witness eight persons affected with it at the Clinical Institution alone; whilst the real cause of the disease has, to the present time, been enveloped in obscurity. I may, therefore, be permitted to advance a new opinion respecting it, which will, perhaps, not be devoid of utility in its application to practice.

It is observed, that for several years past stone in the bladder has become less common than it was formerly, even in the countries where it was once very frequent. This has been attributed, and, I believe, with propriety, to the more general use of diuretic drinks, especially of tea and coffee. Now, the production of saccharine matter in the kidneys, seems to augment in the same ratio as stone diminishes. Does not this phenomenon indicate an analogy between the causes, as well as the nature, of those two diseases? and may not the production of sugar in diabetes be a modification of lithic production? We find in both diseases a similar fundamental condition, an abundance of free uric acid: the only difference is, that in one of these affections the acid unites with mucus, or with earthy particles, to form a calculus; and that in the other, the acid unites with some other substance so as to form sugar. An important circumstance in support of this notion, is, that chemical analysis shews that uric acid, which constantly exists in the urine of a person in the state of health, is not found in that of a person having diabetes. Consequently, after all the fruitless attempts which have been made to cure diabetes, it remains to be examined, whether we should not treat this affection as we treat calculous disorders; and, if the same lithontriptics, which are evidently efficacious against stone and gravel, such as potash, soda, soap, lime-water, and especially the waters of Carlsbad, may not be equally efficacious against the diabetes mellitus.—*Journal der Practischen Heilkunde.*

